# The molecular signature of degeneration in explanted decellularized allogeneic heart valves

**DOI:** 10.3389/fcvm.2026.1865343

**Published:** 2026-07-30

**Authors:** Allison D. Seidel, Julia Rückoldt, Christopher Werlein, Mareike Knoll, Christina Petzold, Regina Engelhardt, Christian Riehle, Alexander Horke, Dmitry Bobylev, Murat Avsar, Samir Sarikouch, Lavinia Neubert, Jan C. Kamp

**Affiliations:** 1 Institute of Pathology, Hannover Medical School, Hannover, Germany; 2 Department of Cardiothoracic, Transplant, and Vascular Surgery, Hannover Medical School, Hannover, Germany; 3 Biomedical Research in Endstage and Obstructive Lung Disease Hanover (BREATH), German Center for Lung Research (DZL), Hannover, Germany; 4 Department of Respiratory Medicine and Infectious Diseases, Hannover Medical School, Hannover, Germany; 5 Department of Cardiology and Angiology, Hannover Medical School, Hannover, Germany

**Keywords:** allograft, decellularization, gene expression, homograft, tissue engineering, valve disease

## Abstract

**Objective:**

The objective of this study was to gain insight into the molecular mechanisms leading to early degeneration of decellularized homografts.

**Methods:**

Formalin-fixed and paraffin-embedded tissues from fresh explanted decellularized aortic (*n* = 7) and pulmonary (*n* = 8) valves were used. RNA was isolated and analyzed using panel-based transcriptomics, focusing on fibrosis- and inflammation-related genes. Differentially expressed genes were used as input parameters for biological pathway analysis using the Gene Ontology Biological Process and Hallmark databanks. Formalin-fixed and paraffin-embedded tissues from freshly explanted healthy donor aortic (*n* = 7) and pulmonary valves (*n* = 8) were used as controls.

**Results:**

Our analysis revealed 56 differentially expressed genes in decellularized aortic valves compared to donor aortic valves, of which 21 and 35 were up- and downregulated, respectively. Decellularized pulmonary valves showed 115 differentially expressed genes compared to donor pulmonary valves, 66 of which were up- and 49 downregulated. In both decellularized aortic and pulmonary valve explants, we found increased expression of fibrosis-, inflammation-, and endothelium-related genes and a decreased expression of genes encoding for complement factors in line with the observed biological pathway activity patterns.

**Conclusion:**

We present a comprehensive transcriptome analysis of explanted decellularized heart valve homografts providing insights into the biological processes leading to continued degeneration and ultimately loss of function. The degeneration of decellularized homografts is driven by inflammation, fibrosis, and extracellular matrix (ECM) remodelling, reinforced by ongoing oxidative stress rather than by a mainly complement-driven humoral immune response.

## Introduction

Tissue engineering of decellularized human donor heart valves has become an established source of grafts (1). The first systemic procedure for decellularization was developed in 1984 and the first clinical application in a pediatric patient was performed in 2002 (2, 3).

The concept of preserving the cell-free extracellular matrix (ECM) of human aortic and pulmonary heart valves constitutes a biological alternative to the replacement of degenerated heart valves by porcine valves or mechanical valve prostheses and is associated with excellent long-term outcomes in adults and children (4). In contrast to the implantation of mechanical heart valves, no lifelong anticoagulation is required making the use of homografts attractive, especially for younger and female patients who desire to have children (5). The implantation of xenogeneic heart valves represents another concept, in which no therapeutic anticoagulation is required. However, these implants show a limited long-term durability (6). Especially in children, xenogeneic heart valves are associated with a higher immune response compared to adults (7). In addition, pronounced early degeneration of cryo-preserved homografts has been reported in younger patients (<20 years) (8). As summarized by Shaddy et al., young patient age can be considered as one of the biggest predictors of implanted pulmonary and aortic allograft valve dysfunction with a decrease in risk from early childhood to adolescence (9, 10). Sarikouch et al. observed similar tendencies in the long-term performance of decellularized pulmonary homografts in children (4). Vogel et al. discussed the limitations of decellularized homografts in neonates and infants (1) and Horke et al. clearly demonstrated that degeneration of decellularized aortic homografts disproportionally increases in younger patients (11).

Previous studies mainly focused on the immune-mediated degeneration of explanted decellularized homografts. However, a typical T-cell-mediated immune response, which had been hypothesized, could not be confirmed (11). Despite the nearly total cell elimination, a residual immunogenicity appears to be present in decellularized homografts (12). To date, no specific antigens initiating an immune response in decellularized homografts have been identified (11). The aim of this study was to gain novel insights into the biological processes and molecular pathways that drive the degeneration of implanted homografts in children and adolescents through transcriptome profiling and immunostaining of explanted homografts.

## Materials and methods

### Study cohorts

Formalin-fixed, paraffin-embedded (FFPE) samples from all decellularized allogeneic heart valves that were explanted until 11/2022 were extracted from the archive of the Institute of Pathology at Hannover Medical School. Initially, a total of *n* = 17 aortic valve and *n* = 16 pulmonary valve homografts were identified for this analysis. A decalcification protocol had to be performed prior to paraffin embedding in the majority of cases. However, following this decalcification procedure, *n* = 10 aortic valve and *n* = 8 pulmonary valve samples had to be excluded due to substantial decalcification-associated mRNA degradation lowering the number of available aortic and pulmonary valve homografts to *n* = 7 aortic and *n* = 8 pulmonary valves. Explanted aortic and pulmonary homografts were compared to aortic (*n* = 7) and pulmonary (*n* = 8) valves from resected heart specimens. Clinical data on all utilized tissues are displayed in Table 1.

**Table 1:** Clinical and histopathological information.

Heart valve	Sex	Age at explantation	Retention period of heart valve	Histology	Analysis
PV	M	2 years, 10 months	11 months	Re-cellularization and re-endothelialization, myxoid degeneration	F + I
PV	M	5 years, 11 months	3 years, 11 months	Necrosis, calcification	F
PV	M	6 years, 9 months	3 years, 2 months	Fibrosis	F
PV	F	9 years	8 years, 3 months	Re-cellularization, foreign-body reaction	F + I
PV	F	12 years	12 years	Re-cellularization and re-endothelialization, sclerosis, myxoid degeneration	F + I
PV	M	16 years	10 years	Re-cellularization and re-endothelialization, myxoid degeneration, calcification, heterotopic ossification	F + I
PV	M	20 years	3 years, 2 months	Degeneration	F + I
PV	M	21 years	15 years	Sclerosis, calcification, granulocytic reaction	F + I
AV	M	1 year, 4 months	4 month	Focal coagulation, calcifying sclerosis, myxoid degeneration	F + I
AV	M	4 years, 11 months	4 years, 7 months	Fibrosis	F + I
AV	M	7 years, 5 months	5 years, 9 month	Myxoid degeneration, scarring, calcification	F + I
AV	M	7 years, 10 months	1 year, 2 months	Sclerosis, myxoid degeneration	F + I
AV	M	13 years	2 years	Re-cellularization, calcification, scarring	I
AV	M	24 years	7 years, 3 months	Calcification, scarring	F + I
AV	M	26 years	9 years	Degeneration, foreign-body reaction	F + I

PV, pulmonary valve; AV, aortic valve; M, male; F, female; F, fibrosis panel; I, inflammation panel;.

Our study was approved by the ethics committee of Hannover Medical School under the ethics vote no. 7872-BO-S-2018. All patients have given informed consent.

### Sample preparation

Explanted decellularized aortic and pulmonary valves were pretreated using a decalcification protocol. Valves were exposed to formic acid for at least 24 h to solve calcifications. In case of persisting severe calcification, the procedure was extended to additional 24 h before FFPE preservation was conducted. This procedure is highly efficient, however, often accompanied by a degradation of biomolecules to varying extents, thereby complicating mRNA sequencing and immunohistochemistry analysis (13, 14). The mRNA was isolated from four to eight 10 μm FFPE slices using the Qiagen RNeasy FFPE Kit (Qiagen, Hilden, Germany), eluted in 22 μl RNase-free water, and mRNA content was assessed using the Qubit RNA IQ Assay (Thermo Fisher Scientific, Waltham, MA, USA). Only samples with a minimum of 200 ng of mRNA were analyzed.

### Transcriptome profiling

Transcriptome analysis was performed using the Nanostring® nCounter analysis platform (NanoString Technologies, Seattle, WA), the human fibrosis V2 panel including 770 human fibrosis-related target genes, and the human inflammation panel including 255 human inflammation-related target genes. Normalization of counts was performed using the nSolver analysis software version 3.0 (NanoString Technologies) using established housekeeping genes contained in the panels. Normalized mRNA counts were statistically analyzed using GraphPad Prism version 9.5.1 for Apple (GraphPad Software, San Diego, CA). Normality was tested using the D’Agostina & Pearson test and comparisons between groups were performed using the Mann–Whitney U-test. Correction for multiple comparisons was performed using the Benjamini-Krieger-Yekutieli two-stage step-up method providing false discovery rates (FDR). FDR-values <0.05 were considered as statistically significant. We performed principal component analysis (PCA) and visualization using R software (version 4.3.0) and the factoextra package (version 1.0.7). Analysis was performed on normalized expression data. Individual samples are shown as points coloured by group, with ellipses representing 95% confidence intervals.

### Biological pathway analysis

Gene Set Enrichment Analysis (GSEA) was performed using the R package clusterProfiler (version 4.17.0) (15). Genes were ranked by log2 fold change values. The analysis included Gene Ontology Biological Process (GOBP, from org.Hs.eg.db v 3.22.0) and Hallmark gene sets from the Molecular Signatures Database (MSigDB v2025.1.Hs; obtained via msigdbr version 25.1.1). *P*-value cutoffs were set to 0.2 for Hallmark and 0.05 for GOBP analyses (15).

### Immunohistochemical analysis

FFPE sections (2 μm) of explanted decellularized allogeneic pulmonary and aortic heart valves were used for immunohistochemical staining. The antibodies used were selected based on the mRNA profile of heart valves. Four pulmonary and 4 aortic explanted heart valves as well as 4 pulmonary and 4 aortic healthy heart valves were chosen as controls. As representative markers for the gene groups shown in Figures 2, 3, collagen 3 (COL3), C-X-C motif chemokine ligand 12 (CXCL12), cadherin 5 (CDH5, also known as vascular endothelial cadherin, VE-cadherin), platelet endothelial cell adhesion molecule-1 (PECAM1, also known as cluster of differentiation 31, CD31), and hypoxia inducible factor 1 subunit alpha (HIF-1*α*) were chosen. Information about the antibodies used are portrayed in Table 2. The FFPE sections were deparaffinized and rehydrated using an ascending alcohol series. After the antigen retrieval, the incubation of the antibody was performed for one hour. For detection 3’3 Diaminobenzidin (DAB) was used and afterwards the slices were counterstained using haemalum.

**Table 2:** Target antibodies for immunohistochemistry.

Target	CAT-ID	Host species	Pre-treatment	Dilution	Manufacturer
CDH5	Sc-9989	Mouse	Sodium citrate buffer	1:200	Santa Cruz
COL3	Ab184993	Rabbit	Tris-EDTA buffer	1:100	Abcam
CXCL12	AP20632PU-N	Rabbit	Sodium citrate buffer	1:200	Acris
HIF-1*α*	Ab8366	Mouse	Sodium citrate buffer	1:500	Abcam
PECAM1	760-4378	Mouse	Tris-EDTA buffer	RTU	Cell Marque

CDH5, cadherin 5; COL3, collagen type 3; CXCL12, CXC motif chemokine 12; HIF-1α, hypoxia inducible factor 1 subunit alpha; PECAM1, platelet and endothelial cell adhesion molecule 1; EDTA, ethylenediaminetetraacetate; RTU, ready to use.

## Results

### Clinical information

The cohort of patients of whom explanted decellularized pulmonary heart valves were analyzed comprised of 6 male and 2 female patients with a median age at time of explantation of 11.9 ± 7.9 years (standard deviation; age range 2–21 years). The cohort of patients with explanted decellularized aortic valves comprised of 7 male patients with a similar age range of 1–26 years at the time of explantation (median age at explantation 12.07 ± 9.52 years). Taken together, the majority of patients (87%) was male with a similar age range in both groups.

Overall, pulmonary homografts showed a longer durability of approximately 9.5 years compared to aortic homografts, which were explanted approximately 4.3 years after implantation. Control hearts were collected from our biobank from previous studies and therefore anonymized. The main reasons for homograft explantation were valve stenosis, sclerosis, and insufficiency. In one case, homograft degeneration occurred in the context of double outlet right ventricle (DOVR)/Goldenhar's syndrome. Another patient may have had a history of endocarditis based on clinical and histological findings. Active endocarditis was not observed in any of the homografts included in this study. More clinical information is shown in Table 1.

### Transcriptome profiling

Comparison of decellularized aortic homografts with control aortic valves showed a total of 56 differentially expressed genes (DEGs) of whom 21 were up- and 35 downregulated.

**Figure 1:**
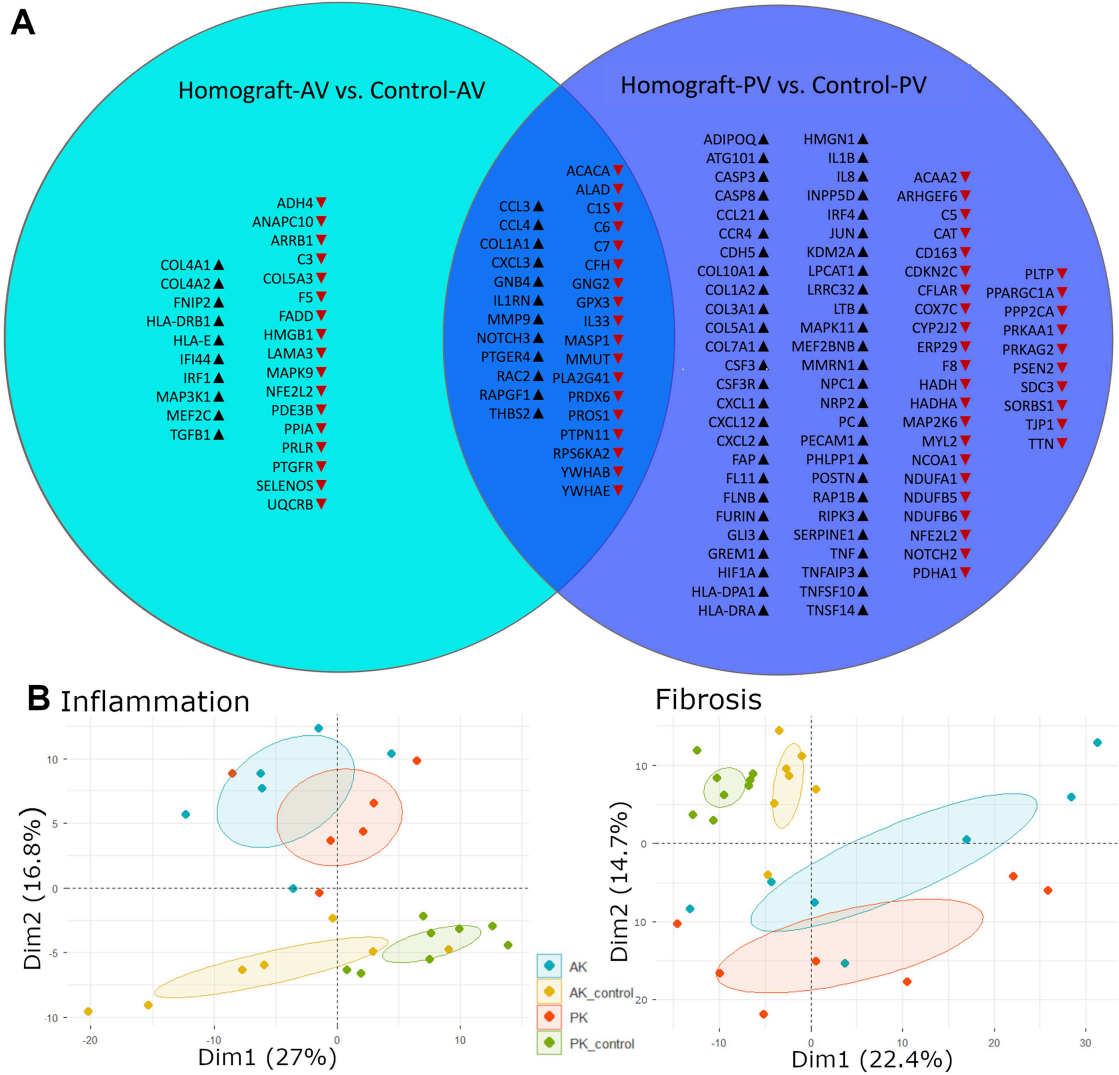
Overview of differentially expressed genes. **(A)** Venn diagram showing differentially expressed genes in decellularized explanted aortic valves (Homografts AV) and pulmonary valves (Homografts PV) compared with respective control heart valves. Increased and decreased expression of genes is indicated by arrowheads. **(B,C)** Principal component analysis (PCA) of the inflammation and fibrosis panel gene sets showing clusters of explanted decellularized pulmonary (PK) and aortic (AK) homografts as well as control heart valves. Each dot stands for one valve.

Comparison of decellularized pulmonary homografts with control pulmonary valves revealed a total of 115 DEGs of whom 66 and 49 were up- and downregulated, respectively. Differential expression between aortic and pulmonary homografts compared to respective controls is visualized in Figure 1. A total of 30 genes were similarly regulated in both decellularized aortic and pulmonary homografts.

**Figure 2:**
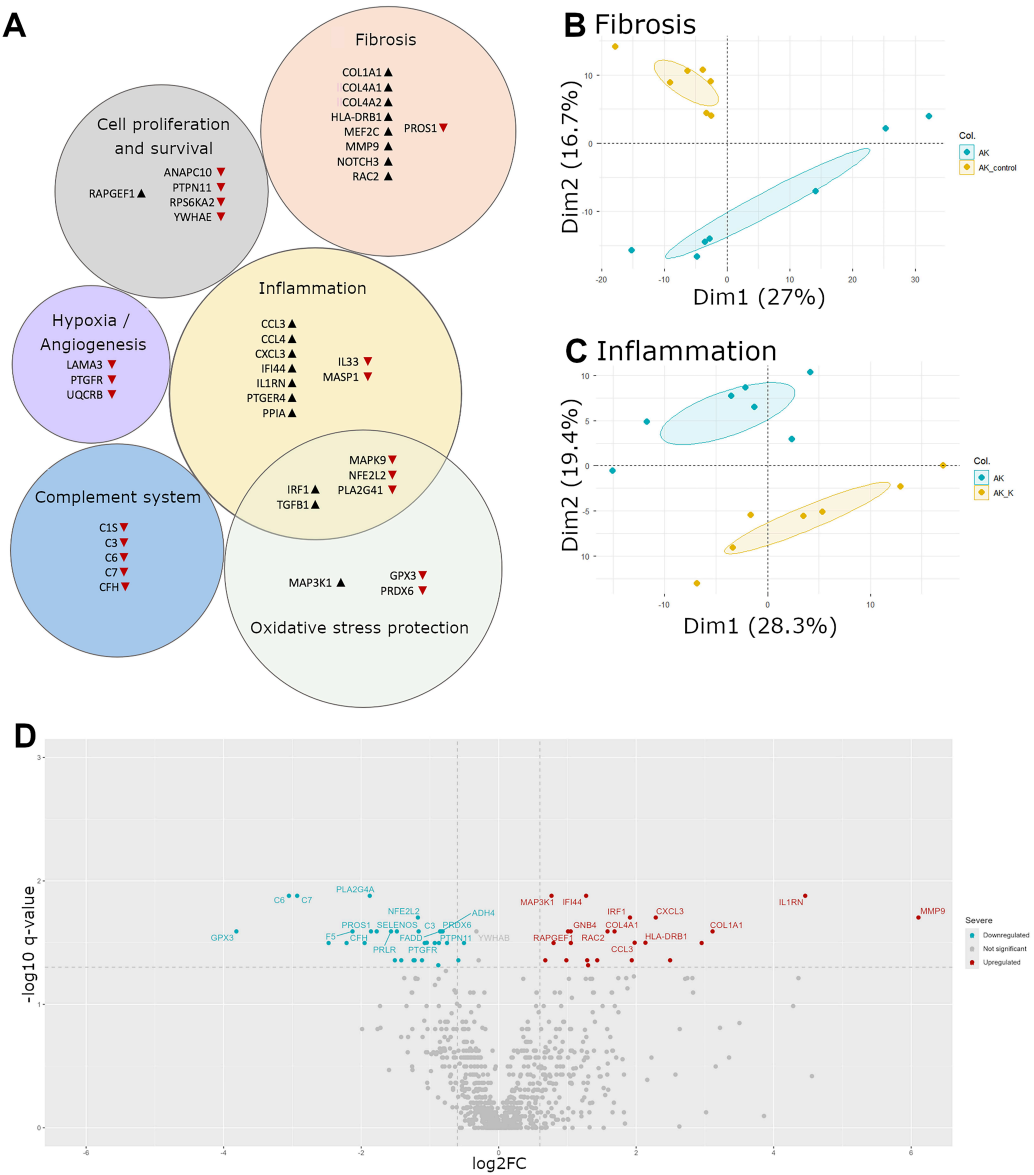
Explanted decellularized aortic valve homografts vs. controls. **(A)** Venn Diagram summarizing differentially regulated genes of explanted decellularized aortic valves compared with control aortic valves classified into functional categories. Increased/decreased transcript levels are indicated by arrowheads. **(B,C)** Principal component analysis (PCA) of the fibrosis and inflammation panel showing clusters of explanted decellularized aortic homografts (AK) and control aortic valves (AK_K). Each dot stands for one valve. **(D)** Volcano plot portraying the top differentially expressed genes of explanted decellularized aortic homografts vs. control aortic valves.

**Figure 3:**
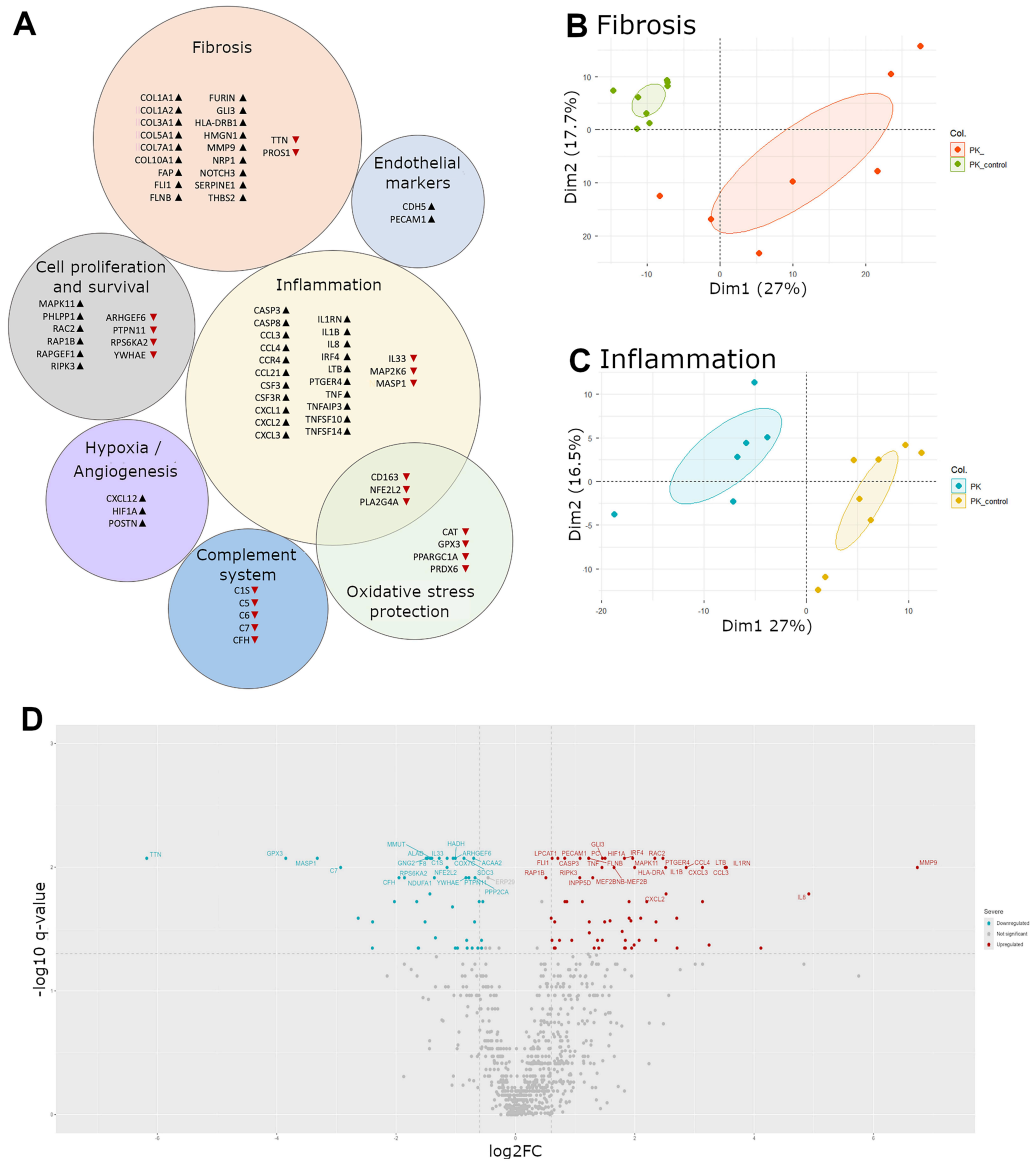
Explanted decellularized pulmonary valve homografts vs. controls. **(A)** Venn Diagram summarizing differentially regulated genes of decellularized explanted pulmonary valves compared with control pulmonary valves classified into functional categories. Up- and down-regulation of genes are indicated by arrowheads. **(B,C)** Principal component analysis (PCA) of the fibrosis and inflammation panel showing clusters of explanted decellularized pulmonary homografts (PK) and control pulmonary valves (PK_control). Each dot stands for one valve. **(C)** Volcano plot portraying differentially expressed genes of explanted decellularized pulmonary homografts vs. control pulmonary valves.

DEGs were categorized into 7 functional groups based on our literature search i.e., complement system, cell proliferation, oxidative stress, inflammation, endothelial markers, hypoxia and angiogenesis, and fibrosis (Figures 2, 3).

Principal component analysis showed a clear clustering of each group while comparing aortic homografts with control aortic valves (Figure 2) and pulmonary homografts with control pulmonary valves (Figure 3). Volcano plots of Figures 2, 3 illustrate the top up- and down-regulated DEGs in pulmonary and aortic homografts.

Many of the top-upregulated DEGs in pulmonary homografts belonged to the inflammation group including cytokines such as TNF-α, C-X-C motif chemokine ligand 3 (CXCL3), and C-C motif chemokine ligand 3 (CCL3). Other upregulated DEGs were related to fibrosis [e.g., matrix metallopeptidase 9 (MMP9), collagen type 3 alpha 1 chain (COL3A1), and collagen type 1 alpha 1 and alpha 2 chain (COL1A1, COL1A2)].

Additionally, typical endothelial markers such as PECAM1/CD31 and CDH5/VE-cadherin and the hypoxia- and angiogenesis-associated gene hypoxia inducible factor 1 subunit *α* (HIF-1*α*) were up-regulated. In contrast, genes related to the complement system such as complement factor C5 (C5), complement factor C6 (C6), complement factor C1s (C1S) as well as genes associated with oxidative stress protection like catalase (CAT) and glutathione peroxidase 3 (GPX3) were down-regulated in pulmonary homografts.

In aortic homografts, TGF-*β* was among the top up-regulated DEGs together with inflammation-related genes like CXCL3. In addition, matrix metallopeptidase 9 (MMP9), a key player in vascular and ECM homeostasis and fibrosis, was up-regulated and showed the highest fold change value. Moreover, mitogen-activated protein kinase 1 (MAP3K1) was prominently up-regulated.

Like in the pulmonary homograft group, reduced transcript levels were observed in aortic homografts for several components of the complement system like C3 and C6 as well as for hypoxia- and angiogenesis-related genes like laminin subunit *α* 3 (LAMA3) and prostaglandin F receptor (PTGFR). In addition, selenoprotein S (SELENOS) and phospholipase AS Group IVA (PLA2G4A) were down-regulated in aortic homografts.

### Biological pathway analysis

Complementary biological pathway analysis revealed several pathways significantly regulated in aortic and pulmonary homografts compared to respective controls as shown in Supplementary Figure 1. Both aortic and pulmonary homografts showed a regulation of various inflammation-related pathways such as TNF-α signaling via NF*κ*B, inflammatory response, immune response, immune system process, response to cytokine, and IL6-JAK-STAT3 signaling as well as of fibrogenesis-related pathways such as epithelial-to-mesenchymal transition, ECM organization, extracellular structure organization, and external encapsulating structure organization. In aortic homografts, additional inflammation-related pathways such as interferon-*γ* response and hypoxia-associated pathways were regulated as well as pathways associated with tissue remodelling such as anatomical structure morphogenesis and cell migration.

### Protein expression analysis

**Figure 4:**
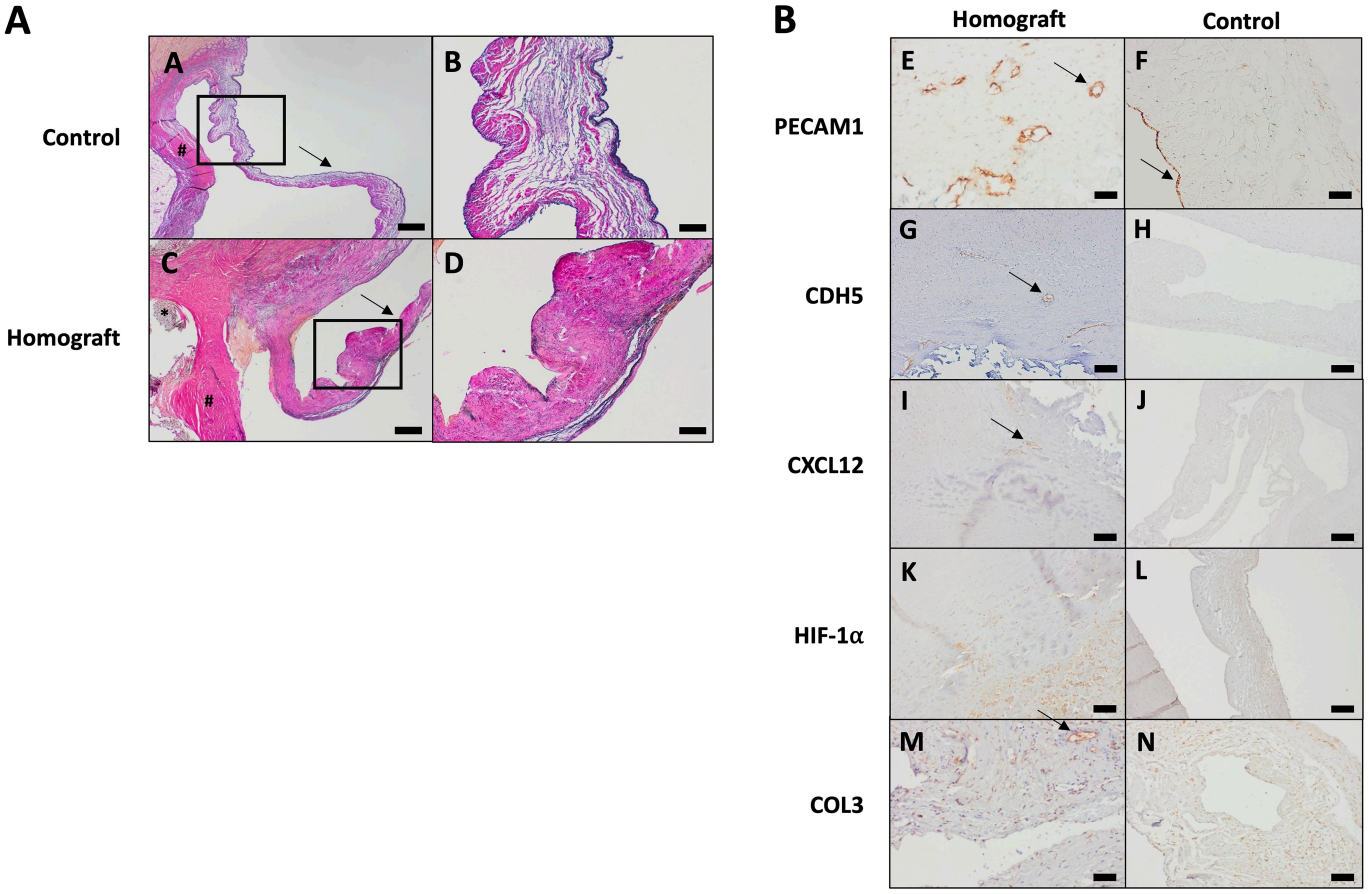
Protein expression analysis. **(A)** Elastica-van-Giesson (EvG) staining of a representative control heart valve (A, framed area magnified in B) and a degenerated homograft (C, framed area magnified in D). Arrows are highlighting the heart valve. **(B)** Immunohistochemical stainings of endothelial markers (PECAM1, CDH5), the chemokine CXCL12, the hypoxia marker HIF-1*α*, and the extracellular matrix protein COL3. Arrows are highlighting small vessels in E, G, I, and M and the endothelial lining in F. Scale bars equal 40 µm in A, J, and L, 20 µm in C, 100 µm in B, D, G, H, I, K, and N, and 200 µm in E, F, and M.

Based on mRNA expression data, the up-regulated genes COL3, CXCL12, CDH5, PECAM1, and HIF-1*α* were selected for immunohistochemical staining to confirm the translational relevance of identified genes (Figure 4). To give an overview of structural changes in degenerated homografts compared to control valves, Elastica van Gieson (EvG) staining was performed (Figures 4A–D). Degenerated homografts showed a structural disruption with prominent hypertrophy due to an increase of ECM and loss of layered tissue organization (Figures 4A,B). In contrast, control valves showed thin leaflets and regularly and layered elastic fibers. As indicated on the mRNA level, HIF-1*α*, CXCL12, COL3, PECAM1, and CDH5 showed an enhanced staining in explanted homografts in comparison to healthy controls. The endothelial markers PECAM1 and CDH5 showed strong staining in small vessels in homografts compared to a weak staining in control valves. PECAM1 showed an endothelial lining only in controls while small vessels were not stained. CXCL12 showed a positivity in homograft endothelial cells, which was not observed in control valves. In line with the mRNA expression data, the hypoxia-related marker HIF-1*α* showed a recognizable staining in homografts, whereas the staining was less prominent and less specific in control valves. COL3 revealed a moderately more prominent staining in homografts, while a staining was also detectable in controls.

## Discussion

To our knowledge, this is the first study to systematically assess the molecular signatures of degeneration in explanted decellularized aortic and pulmonary homografts, with the aim to explore the molecular mechanisms behind graft failure. These findings were further substantiated by pathway enrichment analysis in both aortic and pulmonary homografts compared to respective controls.

Our results indicate that chronic inflammation, fibrosis, as well as extensive ECM remodelling contribute to the degeneration of decellularized homografts in pediatric patients. Oxidative stress appears to add to ECM damage and may further promote inflammation and fibrosis. The clearly observed downregulation of complement-related genes in both homograft types argues against a humoral immune reaction being a main cause of degeneration.

We identified both shared and valve-specific expression patterns in explanted pulmonary and aortic homografts, indicating that shared mechanisms promote graft degeneration while physiological differences exist between both graft types.

One core finding was the consistent up-regulation of pro-inflammatory genes and corresponding proteins in both homograft types compared to respective controls such as TNF-α and CXCL12. In line with these findings, biological pathway analysis showed a regulation of numerous inflammation-related pathways in both homograft types compared to respective controls. There is little evidence on the role of inflammatory processes in homograft degeneration. Sayk et al. reported an early gradual cellular infiltrate in a pulmonary homograft 5 weeks after implantation, which was interpreted as early non-specific inflammation without a specific immunological interference (16). Similar findings have been reported in studies of pulmonary homografts, where no significant cellular immune response could be measured in the recipients’ blood, although this study was performed mainly on recipients with no evidence of homograft degeneration (17).

In contrast, our results indicate that a continued local inflammatory process may drive the degeneration of homografts over time in line with previous reports1. In addition, experimental studies have highlighted the role of CXCL12 on arteriosclerosis based on its production in endothelial cells (18).

According to these findings, the observed positive staining of CXCL12 in endothelial cells of our homograft samples may indicate its involvement in inflammation and remodelling processes of homograft degeneration.

The pronounced presence of endothelial markers like CDH5 and PECAM1 (19, 20) on homografts was an expected result as homografts undergo a process termed “endothelialization” after implantation, i.e., the recellularization of decellularized heart valves with endothelial cells.

In addition, we observed a consistent down-regulation of genes encoding for components of the complement system in both homograft types. Although a predominantly antibody-mediated homograft failure was hypothesized in previous studies (12), our findings question the idea of a chronic complement-driven humoral immune reaction as a chronic damage mechanism in this context. Chronic inflammation leads to myofibroblastic and osteogenic activation and consecutive interstitial cell activation thereby promoting calcific valve disease in both bioprosthetic and homograft valve replacement (21). The sequence between continued inflammation, fibrogenesis, and subsequent calcification has also been reported in another study focusing on the sortilin gene (SORT1) as a key driver (22). Although SORT1 was not included in the gene panel utilized in this study, these data at least support the relevance of this pathophysiological sequence for the degeneration of homografts.

Our results support the present hypothesis that so-called matrikines, specific ECM peptides, which become exposed following processing, may serve as targets for the immune system (23).

In explanted decellularized homografts, we found a significant up-regulation of numerous genes encoding for ECM proteins, like various collagens and MMP9, a protease involved in ECM degeneration and an important generator of matrikines (24, 25). Consistent with these findings, biological pathway analysis revealed a pronounced regulation of ECM-related pathways further supporting the idea of ongoing ECM remodelling in degenerating homografts.

We found a number of up-regulated genes associated with oxidative stress like myocyte enhancer factor 2C (MEF2C) and mitogen-activated protein kinase 1 (MAPK1) (26, 27) as well as down-regulated genes linked to protection against oxidative stress such as GPX3 and peroxiredoxin 6 (PRDX6) (28) indicating that oxidative stress plays an important role in homograft degeneration. It is known that oxidative stress can modify the ECM through protease-based degeneration (29, 30), which may in turn lead to the release of bioactive ECM components - the above described matrikines - thereby again contributing to inflammation and fibrogenesis (31). Our immunohistochemical staining of HIF-1*α* together with the regulation of the associated signaling pathway in homografts support this theory.

Although there was a significant overlap between DEGs of pulmonary and aortic homografts, some differences were detected in our study. Pulmonary homografts showed an up-regulation of several collagen genes compared to aortic homografts while aortic homografts demonstrated a more pronounced up-regulation of inflammation-associated genes and related pathways compared to pulmonary homografts. There was also an enrichment of pathways like “anatomical structure morphogenesis” and “cell migration” in aortic homografts. One might speculate that the differences found between aortic and pulmonary homografts may correlate with haemodynamic factors, i.e., pressure differences between the pulmonary and the systemic circulation (31), supporting the idea that higher mechanical stress in the aortic position activates more inflammation-related pathways while the lower mechanical stress in the pulmonary position contributes to remodelling-related changes (32). Another aspect to consider is that aortic homografts were thicker and had a more complex matrix than pulmonary homografts (33). These properties may limit antigen removal, particularly in aortic homografts (1, 34).

In summary, we here present a comprehensive transcriptome analysis of explanted decellularized heart valve homografts and provide insights into the biological processes leading to continued degeneration and ultimately loss of function of these homografts. We identified distinct pro-inflammatory and pro-fibrotic genes and signalling pathways as key drivers of chronic homograft degeneration and revealed a role of matrix-centered remodelling in this context.

This study is limited by the monocentric design and by the small sample-size, which is a result of the aggressive decalcification protocol necessary for the work-up of this rare resource, as some homografts had to be excluded due to substantial mRNA damage. In addition, the validity of our protein expression results is also slightly limited due to the decalcification protocol, which may harm protein integrity. Another limitation is the lack of ideal controls (i.e., unused homografts). Future studies in larger cohorts including measurements of circulating markers that correlate with the here identified tissue markers are needed to validate these data and to potentially explore the effect of anti-inflammatory and anti-fibrotic therapies.

## Data Availability

The datasets presented in this study can be found in online repositories. The names of the repository/repositories and accession number(s) can be found below: https://doi.org/10.6084/m9.figshare.32051796.
