# Supplementary material for: The molecular signature of degeneration in explanted decellularized allogeneic heart valves
Source: Front Cardiovasc Med. 2026 Jul 30;13:1865343. doi: 10.3389/fcvm.2026.1865343 (PMC13470892; doi:10.3389/fcvm.2026.1865343)
Supplement: Supplementary file 1 [file Datasheet1.pdf]

Supplementary Material

Supplementary Figure 1: Comparison of gene expression in pulmonary and aortic valves

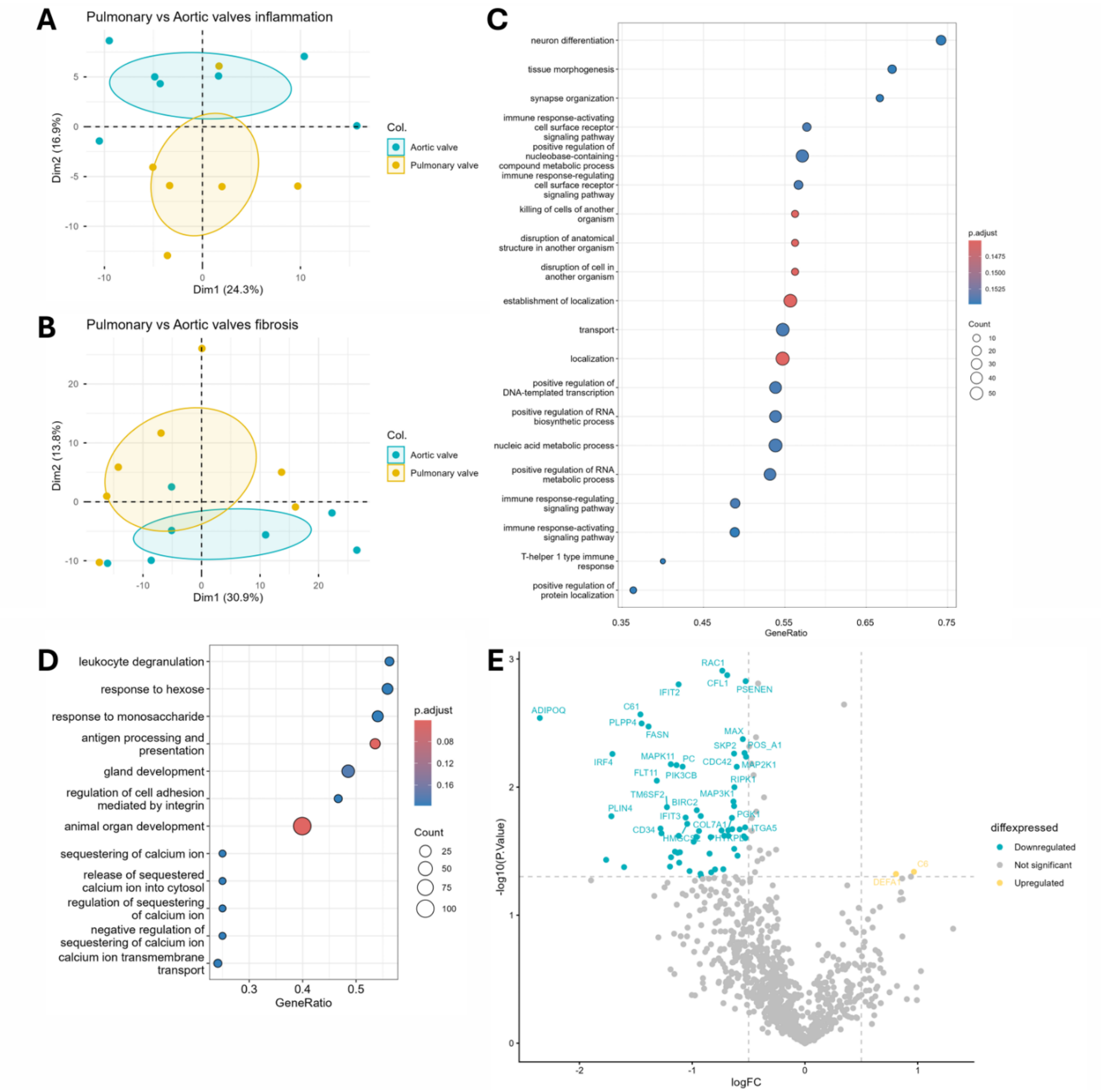

Direct comparison of genesets measured in explanted decellularized aortic vs. pulmonary heart valves showed a clear clustering of groups (A, inflammation panel and B, fibrosis panel). Biological pathway analysis (C, inflammation panel and D, fibrosis panel) as well as differential gene expression analysis of the entire geneset showed differences between groups. However, based on these results, it cannot be distinguished if these differences are due to the localization of implanted homografts with differing hemodynamic conditions or represent specific pathophysiological features.

Supplementary Figure 2: Trajectory analysis of representative markers

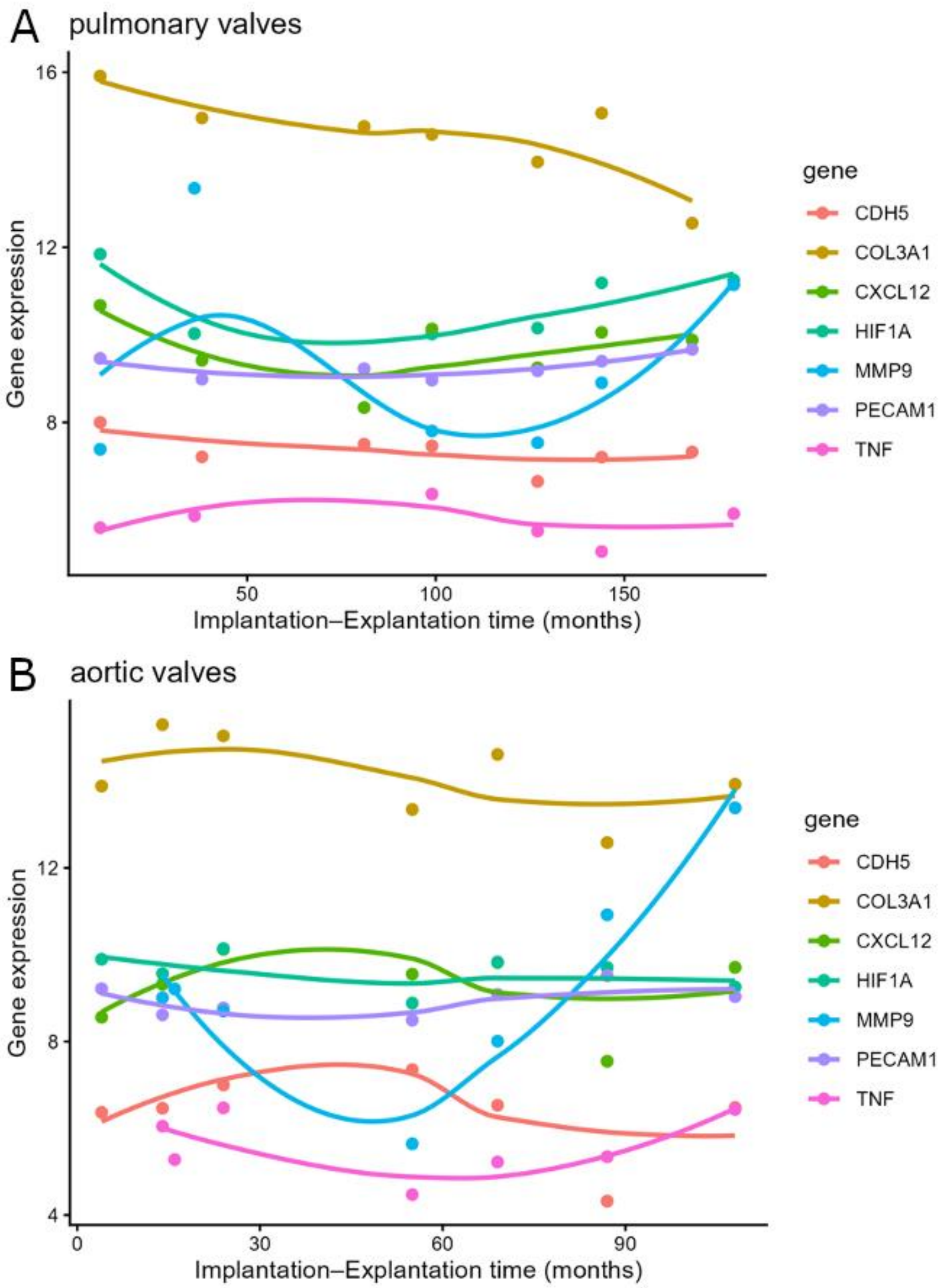

Given that the implantation duration of decellularized heart valves differed significantly between recipients, a trajectory analysis was performed to exclude confounding effects that correlate rather with implantation duration than with degeneration. Log2 gene counts were displayed over time. However, no implantation duration-dependent increases or decreases could be identified indicating that the expression level of these markers does not correlate with implantation duration.

Supplementary Figure 3: Gene Set Enrichment Analysis (GSEA)

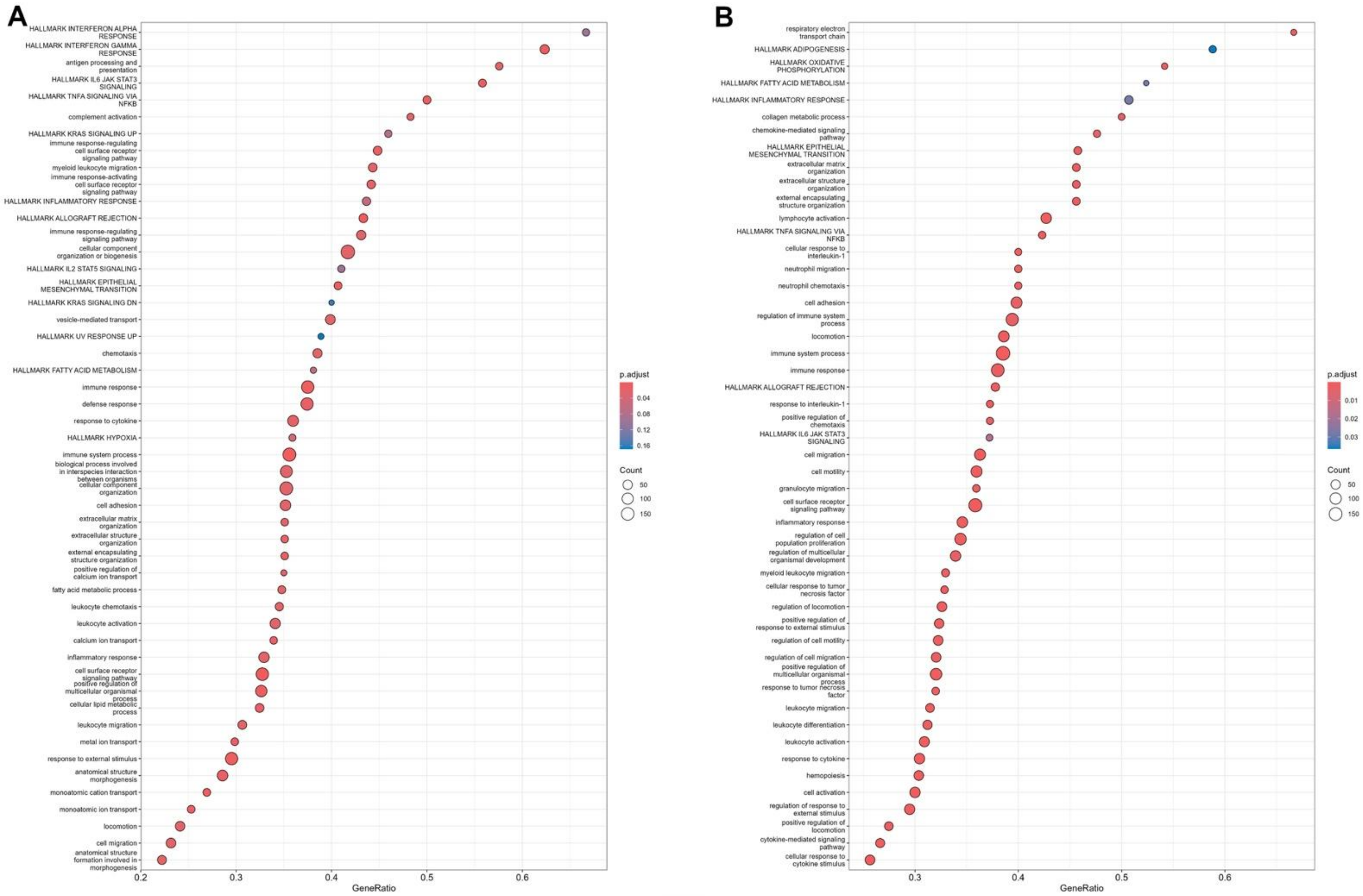

Significantly regulated pathways of aortic (left) and pulmonary (right) homografts in comparison to respective healthy controls. Vertical listings equal the names of databanks used (GO and KEGG) and horizontal graphs show the gene ratio. The circle size stands for the gene counts and the colour intensity represents the adjusted p-value.
